# The Interplay between Microscopic and Mesoscopic Structures in Complex Networks

**DOI:** 10.1371/journal.pone.0021282

**Published:** 2011-08-01

**Authors:** Jörg Reichardt, Roberto Alamino, David Saad

**Affiliations:** 1 Complexity Sciences Center, University of California Davis, Davis, California, United States of America; 2 Institute for Theoretical Physics, University of Würzburg, Würzburg, Germany; 3 The Nonlinearity and Complexity Research Group, Aston University, Birmingham, United Kingdom; Indiana University, United States of America

## Abstract

Understanding a complex network's structure holds the key to understanding its function. The physics community has contributed a multitude of methods and analyses to this cross-disciplinary endeavor. Structural features exist on both the microscopic level, resulting from differences between single node properties, and the mesoscopic level resulting from properties shared by groups of nodes. Disentangling the determinants of network structure on these different scales has remained a major, and so far unsolved, challenge. Here we show how multiscale generative probabilistic exponential random graph models combined with efficient, distributive message-passing inference techniques can be used to achieve this separation of scales, leading to improved detection accuracy of latent classes as demonstrated on benchmark problems. It sheds new light on the statistical significance of motif-distributions in neural networks and improves the link-prediction accuracy as exemplified for gene-disease associations in the highly consequential Online Mendelian Inheritance in Man database.

## Introduction

Networks are fascinating objects. Charting the interactions between system constituents, abstracted as edges and nodes, has allowed us to marvel the interconnectedness of systems and appreciate their complexity. Whether in foodwebs [1], social communities [2], protein-interaction [3], metabolism [4], neural networks [5] or communication [6], the network-metaphor has been highly successful in advancing our understanding of complex systems. Many insights were obtained through rigorous analysis and modeling of network structure. In fact, a primary goal of network research is to infer unobserved, or latent, node properties through structural analysis.

One hallmark of complex systems is that they exhibit structure at many scales. In particular, real-world complex networks will generally combine microscopic structural features resulting from single node properties with mesoscopic structural features due to group properties. Separating the two is *essential* for both correctly discovering mesoscopic structures as for inferring single-node behavior. Especially as node characteristics and functions may differ radically among individual nodes sharing the same group properties. To solve this problem, we advocate the use of generative probabilistic modeling and physically motivated inference techniques.

Though the statistical physics community has played a leading role in the cross-disciplinary effort to understand complex network structure [7], most analyses have avoided the problem of disentangling the microscopic from the mesoscopic scale. Rather, they focus on either of the two, explaining network structure from either the microscopic *or* the mesoscopic viewpoint. For example, when modeling degree distributions [6], [8], analyzing the distributions of centrality indices [9] or the distributions of small subgraphs, so-called motifs [10], group effects are rarely taken into account. Conversely, individual node properties are generally neglected in inferring latent node classes from network structure via block structure [11] or community detection algorithms [12]. As a result, one inevitably attributes individual node statistics to the inferred group properties and vice versa, leading to misinterpretation of individual node statistics and their significance on the one hand and inaccuracies in latent class identification on the other.

Here we present a consistent and principled probabilistic approach to the inference of latent node characteristics that allows one to separate the effects on the level of groups of nodes from the level of individual nodes. Specifically, we present a generative probabilistic model for the inference of latent node classes that includes node specific features. The model gives rise to a realistic ensemble of statistically weighted networks matched to an observed dataset, and facilitates the derivation of parameter expectation values and corresponding confidence intervals as well as the differentiation between more and less important structural features. We will show that the combination of node specific and group specific effects in the model allows for a much improved accuracy in the inference of latent classes of nodes. It can shed new light on the assessment of statistical significance of motif distributions in networks and finally, it leads to dramatically improved accuracy in predicting unobserved links as shown using a network of gene-disease associations from the Online Mendelian Inheritance in Man database.

### Exponential random graphs

The probabilistic framework used is that of exponential random graph models (ERGMs) [13], [14] as they exhibit several desired properties: ERGMs are mean unbiased and make the observed data maximally likely; they are maximum entropy models thus ensuring the generated networks are maximally random in all aspects other than those modeled explicitly. In other words, they parameterize the largest ensemble of networks compatible with our observations, while making the observed network typical for the ensemble. Additionally, they have a clear mapping onto the statistical physics framework of spin models and facilitate the combination of node and group specific properties using parameters that have a very intuitive interpretation.

**Figure 1:**
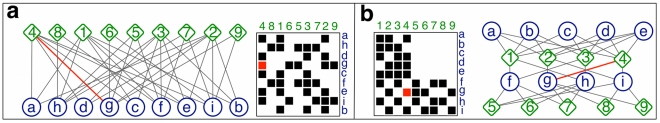
An actor-event network and its adjacency matrix. **a,** In the network, actors are represented as circles, events as diamonds. Links indicate the participation of an actor in an event. In the adjacency matrix, actors are represented by rows and events by columns. A non-zero (non-white) entry in row 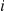, column 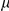 indicates participation of actor 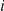 in event 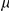. As an example, the edge between event 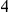 and actor 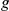 is highlighted in all network representations. Without the knowledge of latent classes for either actors or events, both representations appear unstructured. **b,** The same network as in a, but rows and columns of the adjacency matrix have been reordered, such that blocks in the adjacency matrix become apparent indicating the presence of latent classes of actors and events. We address the challenge of inferring such latent classes through statistical modeling, which leads to assertions of node properties or can generate improved network layouts.

Consider a given, bipartite network specified by an 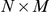 adjacency matrix 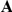, representing for instance the attendance of 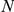 actors in 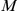 events. If actor 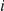 has attended event 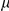, then 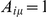 and otherwise 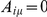. Equally, 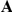 could represent the association of 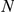 diseases with 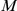 different genes or the choices of 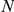 consumers from a list of 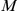 products. The possibilities are many and we will use the actor-event picture, presented pictorially in figure 1, but without limiting the applicability of the model to this case alone.

We restrict ourselves to *dyadic* models, *i.e.* we assume the entries of the adjacency matrix 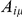 to be modeled by the conditionally independent random variables 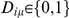. A simple ERGM that captures both individual (actor- and event-specific) and group-specific properties is given in terms of the odds ratio of actor 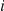 attending event 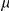:
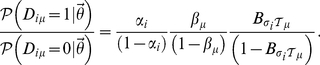
(1)


The shorthand 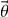 in (1) denotes the set of all model parameters 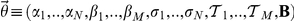. Note how the model assumes a physically interpretable exponential form by rewriting the product of parameters in (1) as 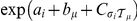 where 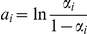, 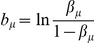, and 
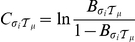
. Interpreting the variables of the model matrix 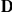 as Ising spin-like variables, the log of the likelihood 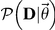 then corresponds to the energy of an Ising spin-like system under the action of external fields 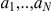, 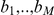 and 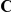. In this parlance, parameter estimation corresponds to determining the external fields that best match 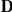 to the observed data 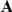.

Of all parameters 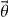 only a small subset is relevant for an individual dyad 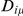 in (1). The parameter 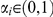 denotes the global *activity* of actor 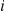, higher 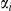 means higher odds of attending any event. Correspondingly, 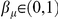 denotes the global *popularity* of event 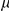. Furthermore, every actor 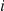 and every event 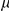 carry a class index 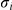 and 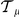, respectively. The number of classes is determined a priori here; it represents a free parameter that defines the coarseness or resolution of the grouping sought. The matrix 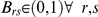, models the data at a coarser, group specific level, denoting the *tendency* or *preference* of an actor of class 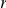 to attend an event of class 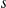. Higher entries mean higher odds for the attendance of any actor of class 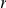 to any event of class 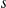. The matrix 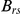 is also called a block model of the data.

The rich literature on ERGMs [15] has generally assumed prior knowledge of the class labels 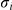 and 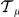 in (1), or other covariates [16]–[19]. Then, learning the parameters of (1) practically reduces to a simple logistic regression. However, the learning task is considerably more complicated if the latent class labels 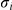 and 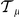 are unknown and need to be inferred. On the other hand, a growing body of work is dedicated to the development of efficient algorithms for learning general stochastic block models [20]–[24] *including* the hidden assignment of nodes into classes, but *without* the incorporation of node specific effects, *i.e.* a model specified by
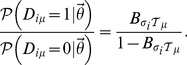
(2)


This model is also referred to, with slight variations, as infinite relational model [25] or mixed membership stochastic block model [26]. Attempts to include the estimation of node specific effects have resulted in biased models [27]–[29]. Within the framework of ERGMs, node and group specific properties have been combined in so called latent space models [30], [31] where nodes are assigned a position in an abstract space and links form as a function of their distance. Such models are well motivated for social networks, where homophily is a central mechanism of link formation and proximity in the latent space may be interpreted as similarity. Yet they are less general than stochastic block models being caught in the predicament of placing groups of nodes with similar interaction partners in close proximity while at the same time having to place them further apart if the nodes are not densely connected.

Our approach facilitates parameter estimates and latent class inference in a principled model (1) which combines node specific effects with the more general stochastic block models for group structure. To estimate model parameters efficiently, we employ distributive message-passing techniques, with computational complexity scaling linearly with the problem size. Generalizing the probabilistic model (1), algorithm and update equations to directed and undirected uni-partite networks is straightforward with some modifications. Most notably, in directed uni-partite networks, represented by an 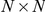 adjacency matrix 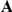, dyads are represented by 4-state variables 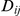 to account for all possible directed connections between nodes 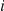 and 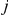. Further, directed networks necessitate the introduction of a reciprocity parameter that explicitly models the co-occurrence of a link from 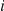 to 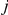 and 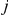 to 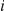. In the analysis presented here, we have allowed for reciprocities to vary depending on the latent classes of nodes. Details of the inference method used can be found in the Methods section and Material S1.

## Results

Using three dedicated examples, we compare the effects of combining microscopic (node specific) with mesoscopic (group specific) effects as in model (1) versus including only one of the two scales.

### Southern Women

First, we demonstrate the impact of including microscopic (node specific) effects on inferred mesoscopic latent class structure. To this end we compare model (1) with the less expressive standard stochastic block model (2) using a dataset from sociology. This classic bipartite data set is due to ethnographers Davis, Gardner and Gardner [32]. A 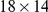 matrix records the attendance of 18 women in southern Alabama to 14 informal social events over the course of a nine month period in the 1930s. The authors' aim was to study how an individual's social class influences her pattern of informal social interaction. Based on intuition and experience in the field, but without formal analysis, the authors suggested the existence of two latent classes of 9 women each, with only little overlap in the attendance at events. Over the years, the data has become a standard test case of network analysis algorithms, a meta-analysis of which can be found in [33]. We are interested in whether an inference based approach can assert the presence of latent social classes and whether the class assignments found correspond to those suggested by the experts.

**Figure 2:**
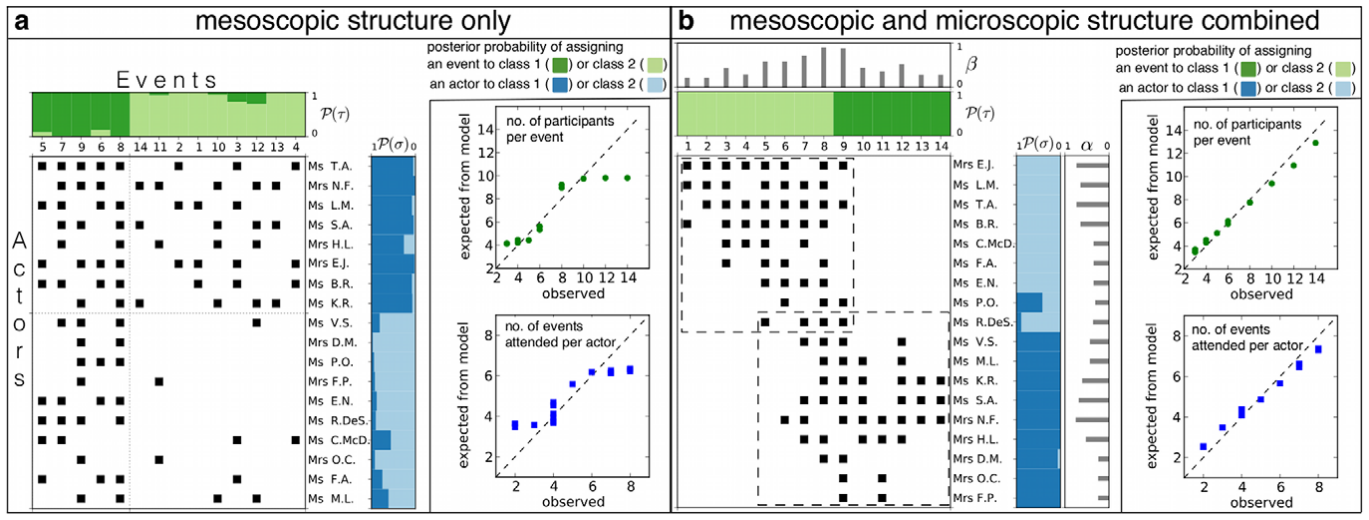
Attendance record of 18 women (rows) to 14 informal social events (columns), black squares indicate attendance. **a**) Attendance matrix with posterior probability of class assignment for actors 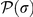 and events 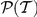 as found by learning a standard stochastic block model (2). Classification inferred divides events according to number of attendants and actors according to the number of events participated in. The Inset shows the observed numbers of attendances do not agree well with the expectations due to model (2). **b**) The same attendance matrix as in a) but reordered due to the classification given in the original study indicated by dashed boxes [32]. Posterior probability of class assignments inferred using model (1) is almost perfectly compatible with the expert's classification. Including node specific popularity and activity parameters 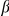 and 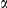 allows to match observed numbers of attendances vs. expectations from model (1) as shown in inset.

If the network's structure could be explained entirely due to a latent (social) classes, the standard stochastic block model (2) should be able to capture it. Allowing for two classes of actors and events, as suggested by the original authors, we learn the standard stochastic block model and estimate class membership 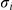, 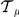 and preference matrix 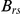. Figure 2a shows the data, with rows and columns of the attendance matrix reordered such that events/actors predominantly assigned to the same class are adjacent. The resulting block model is in stark contrast to findings of the original authors [32]. Events seem divided according to the number of participants (popularity) while actors seem divided according to the number of events participated in (activity). The expert classification due to social class is not correctly captured when trying to model the network through group effects alone. The reason is that under model (2), the degree distribution for members of the same latent class is assumed to be Poissonian. The expected degree is the same for each member of a given class. The inset in figure 2a shows that this assumption cannot capture the observed degree distribution. Since the standard stochastic block model does not model node degree independently of class preference; variance in degree distributions of both actors and events confuses the inference of group membership.

In contrast, the inset in figure 2b shows the expected degree vs. the observed degree when activity and popularity parameters are included in the model (1) and allowing for two classes. Now, the observed degree distribution can be accounted for. The introduction of activity and popularity parameters has also dramatic effects on the latent classes inferred. Figure 2b shows the attendance matrix, where rows and columns are ordered as given in [32] and the authors' assignment to social class is indicated by dashed boxes. The experts' classification matches almost perfectly that inferred using model (1). We can see that events such as 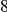 and 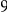 which are attended by most actors receive high 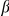 values and thus have very little discriminative power. Also, actors who are very active and occasionally participate in events predominantly frequented by actors from the other group, such as Mrs. N. F., can still be assigned with high probability to a class, despite conflicting evidence in their participation record. Using model (1) effectively allows one to decouple the preference effects of a group of actors for a group of events from global effects that contribute to the variance in node connectivity.

### Caenorhabditis elegans

Second, we examine the importance of including mesoscopic group effects in the interpretation of microscopic structural features. To this end, we study to which extent a dyadic model may explain the distribution of small sub-graphs (motifs) in the neural network of the nematode *C. elegans*.

Motifs have received considerable attention as possible entities of network formation, *i.e.* building blocks larger than single edges. Their distribution relative to random null models has been suggested to characterize entire classes of networks [10]. The over/under-representation of certain motifs with respect to random null models is often attributed to possible evolutionary pressures due to a motif's potential influence on the performance of the network's function [34],[35].

**Figure 3:**
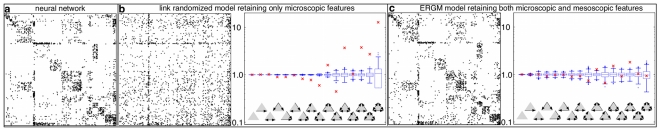
Motif counts in the synapse network of C. elegans compared to two random null models. **a**) Adjacency matrix of the observed neural network [36]. **b**) Adjacency matrix of a typical realization of a link randomized version of the original data and resulting Z-score statistics of motif counts. Counts in the original data (red x) are compared to box plots of counts in 1000 link randomized null models. Strong deviations are found at 11 of the 16 motifs. Since the link randomized null models retain only node specific features, *i.e.* the numbers of incoming, outgoing and reciprocated links at each node, the cannot capture the apparent mesoscopic structure in the original network and hence may over-estimate the statistical significance of some motifs. **c**) Adjacency matrix of a typical network generated from a model similar (1) with both node specific as well as class specific parameters estimated from the original network. 15 classes were used in this example. Using 1000 networks generated from this model as a reference ensemble, the Z-score statistics show mild deviations only at 3 of the 16 motifs. This indicates that class structure may offer a more parsimonious explanation for the observed motif distribution.

We study the distribution of all 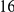 possible 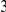-node motifs in the 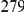 neuron chemical synapse network of *C. elegans* [36]. Figure 3a shows the corresponding adjacency matrix. The null model commonly used to assess whether a particular motif is under- or over-represented in a network is generated by randomizing the original network conserving only microscopic structural features, *i.e.* the number of incoming, outgoing and reciprocated links at each node is preserved. All other structural features and correlations are removed by the randomization. Figure 3b shows one typical adjacency matrix and box-plots for motif counts in 1000 such random networks compared to the actual count of the 16 motifs in the chemical synapse network of *C. elegans*. Counts are normalized to the mean count found in the set of null models. We can see that using such a link randomized null model, 11 of the 16 motifs are strongly over/under-represented and hence would qualify as possible starting points for further research on putative functional relevance.

However, the standard null model also removes all mesoscopic structures, in particular structure due to groups of more than three nodes. The dyadic model which corresponds to (1) lacks any parameter for three-node motifs but can generate an ensemble of null models that matches the observed network in terms of the observed node specific degrees as well as with respect to mesoscopic structural features. Such mesoscopic structure inevitably exists as neurons are located in different somatic regions and synaptic connections between closely located neurons are more likely than between distant ones [37]. Neurons are also aggregated in different ganglia making intra-ganglia connections more likely than inter-ganglia synapses. Furthermore, they serve different functions that influence their connectivity. For example, stimuli may be processed in a sensory neuron - interneuron - motor neuron cascade. The latent classes we infer from the data using the parallel model to (1) can be explained using a combination of these factors (see Material S1 and Dataset S1). More important than the interpretation of these classes is whether a dyadic model, which assumes all pairs of nodes as conditionally independent, can account for the observed three node motif-counts in the network.

Figure 3c shows the box-plots of motif counts in 1000 networks generated from a model similar to (1) allowing for 15 different classes of neurons and using the parameters estimated from the original network, again normalized to the mean count. The comparison with the motif-count in the *C. elegans* network now shows that only 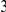 out of 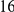 motifs cannot be explained by the null model and deviations from random expectations are much smaller. This result is remarkable as it underscores the importance of group specific effects in modeling complex networks. The fact that a simple dyadic model can explain a large portion of the three-node statistics in the observed data is a strong corroboration for our claim that latent classes of nodes are important determinants of network structure. Furthermore, it offers a very parsimonious explanation of motif statistics in this network and a more conservative estimation of their statistical significance.

### Online Mendelian Inheritance in Man

Third, we determine the predictive ability and classification accuracy of model (1), which accounts for both node and group specific effects, compared to both less and more expressive models. To this end, we study the network of gene-disease associations from the Online Mendelian Inheritance in Man (OMIM) database.

This bi-partite network known as the human “Diseasosome-Network” [38] represents known associations between genes and diseases recorded in the OMIM database [39]. The network was first published in 2005 and we focus on the analysis of the largest connected component involving 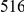 different diseases and 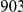 different genes connected by 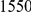 different associations known in 2005 [38] (cf. Dataset S2). The original publication provided an expert classification of the diseases into 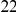 types. The type of disease is predominantly based on the tissues and organs involved (such as bone, connective tissue, muscular, dermatological, hematological, renal, etc.) or based on the affected system (such as skeletal, cardiovascular, immonological, metabolic or endochrinal, etc.).

To what extent does such a classification overlap with one inferred from a network of common genetic causes? We compare model (1) with the less expressive standard stochastic block model (2) and a more expressive model due to Newman and Leicht (NL) [28]. The latter includes both individual and group effects as in (1), but instead of a single parameter for the overall activity or popularity of a node, it features one such parameter per latent class.

**Figure 4:**
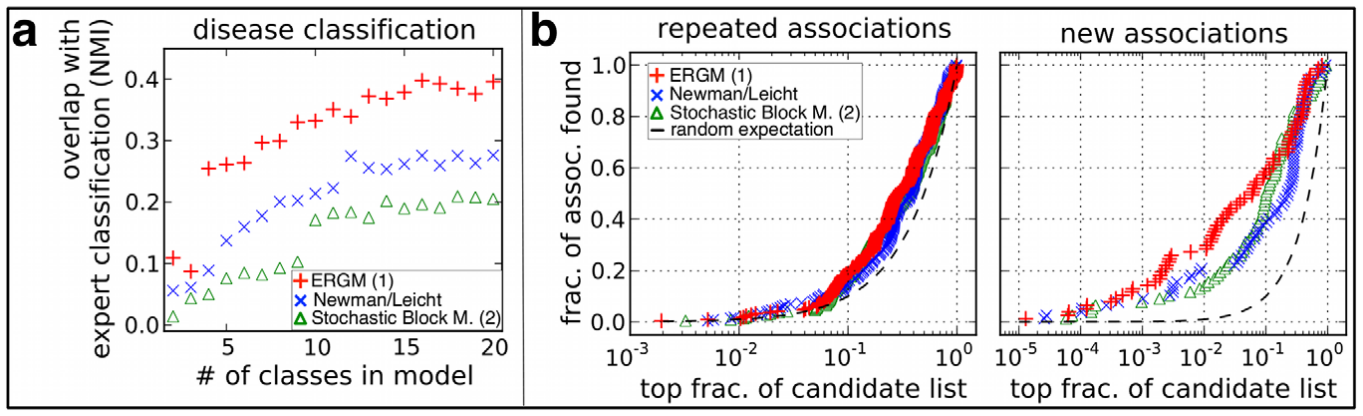
Classification accuracy and predictive power of network models (1), (2) and that by Newman/Leicht (NL) [28]. **a**) Overlap of an expert classification of diseases in the Diseasosome-Network [38] and that inferred using models and the data of known gene-disease associations recorded in the Online Mendelian Inheritance in Man (OMIM) database by Dec. 2005. Measure of overlap is normalized mutual information (NMI) [43]. **b**) Prediction accuracy at 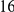 classes for confirmed associations added to the OMIM database between Dec. 2005 and Jun. 2010. For each model, a candidate list of associations is obtained by sorting all possible associations in descending order according to their probability under that model with parameters estimated from the Dec. 2005 data. We plot which fraction of actually confirmed associations is found in the corresponding top fraction of the candidate list. Entries due to new variants of a previously recorded association are listed as “repeated associations” while genuine new associations are reported as “new associations”. For example: In the top 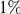 of any candidate list, we expect to find 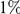 of new associations due to chance alone. We do find 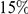 of all confirmed new associations if the list was due to model (2), 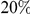 if the list was due to the NL model and 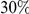 if the list due to model (1). See text for details.

We compare the overlap between the expert classification of diseases and the one found algorithmically, based on the gene-disease association network alone. We restricted ourselves to using the same number of classes for both genes and diseases. The comparison of models (1), NL and the standard stochastic block model (2) is shown in figure 4a. As expected, neglecting individual node effects as in model (2) reduces the overlap with an expert classification compared to model (1). But, interestingly, the same applies when including gene-specific effects for every class of diseases and disease-specific effects for every class of genes as in the NL model. Too many explanatory variables per individual node seem to reduce the detection quality of latent classes.

Since 2005, the OMIM database has been steadily growing and 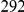 new associations between those 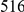 genes and 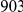 diseases had been added until June 2010. Using the data from 2005 as a training set and these new additions as a test set, we compare the predictive power of the different models for future associations. New entries to OMIM comprise both new variants of already known gene-disease associations (repeated associations) as well as genuine new associations of genes with diseases that were not linked previously. Hence, the data offers the opportunity to differentiate predictive power with respect to these two types of entries (cf. Dataset S3). Using the parameters estimated from the 2005 data set for each model (1), NL and (2), we calculate the probability for association of each gene 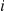 with each disease 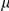 as 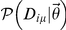. Then we sort these probabilities in descending order and hence obtain a candidate list for new or repeated associations. For instance, in the case of models with 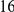 classes (cf. Dataset S4), figure 4b shows how far one has to go down the candidate list to find a certain fraction of the associations that were added to the database over the course of 4 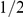 years.

Variants of already known associations seem to be added approximately randomly to the database as models (1), NL and (2) all perform close the random expectation for repeated associations. For the genuinely new associations, however, we observe that all models strongly deviate from the random expectations. In particular (1) outperforms both NL and (2), with the latter two performing similarly.

Figures 4a and 4b show that the generative probabilistic model (1) captures the biologically relevant network structure, offering high classification accuracy and a parsimonious inclusion of node-specific effects, which leads to a superior predictive ability.

## Discussion

We have presented an efficient, distributive algorithm that successfully estimates the parameters and latent group assignments of an exponential random graph model including both node specific and group specific properties. We have shown that including node specific effects in the estimation of latent classes leads to improved recovery of class assignments by domain experts. Additionally, we have shown that including group specific effects in a random null model used to assess the statistical significance of microscopic network motifs may already suffice to explain a large part of the observed motif statistics. This finding sheds new light on the discussion of motif distributions in complex networks and we expect our results to stimulate a discussion on the use of appropriate null models in the analysis of sub-graph distributions and their universality for certain classes of networks. Finally, we have explored the predictive power of the model to identify new gene-disease associations, using the OMIM database. Through these specific examples, we have demonstrated that node specific and group specific properties should be both incorporated when inferring and modeling structural features in complex networks.

## Methods

To describe the probabilistic inference algorithm used for estimating the parameters 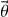, we first write the likelihood of the entire observed network adjacency matrix 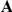 in terms of our model (1):
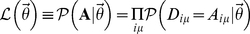
(3)


For a dyadic model, the likelihood factorizes into terms that involve parameters associated with only two nodes.

Commonly used methods to estimate the parameters and hidden variables in such a model are to employ maximum likelihood (ML) techniques in the form of an expectation-maximization type algorithm or Monte Carlo sampling [40]. We prefer a Bayesian approach, based on Maximum A Posteriori (MAP) estimates that does not incur the computational cost of Monte Carlo sampling while being less sensitive to initial conditions and more stable numerically than ML, especially as the parameters which maximize (3) may lie on the the borders of the admissible interval 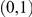. Furthermore, the MAP approach provides a natural Occam's razor as the posterior distributions of parameter estimates can only reduce in variance with the provision of more data, while the ML approach assumes point estimates or 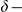
functions for the posterior from the start. This is an important feature of the Bayesian approach as it provides a natural limit for the number of inferred classes and confidence levels in the assignments. Classes cannot be arbitrarily small if the posterior for the inter-class link preference 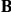 is to be localized. In contrast, under an ML approach the likelihood increases monotonically when more and hence smaller classes are used and model selection criteria, as in [19], are needed. Finally, Bayesian techniques offer a principled way to incorporate prior domain knowledge for obtaining a more accurate approximate marginal posterior distribution 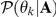, where 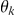 represents one of the parameters 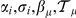 or 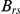.

**Figure 5:**
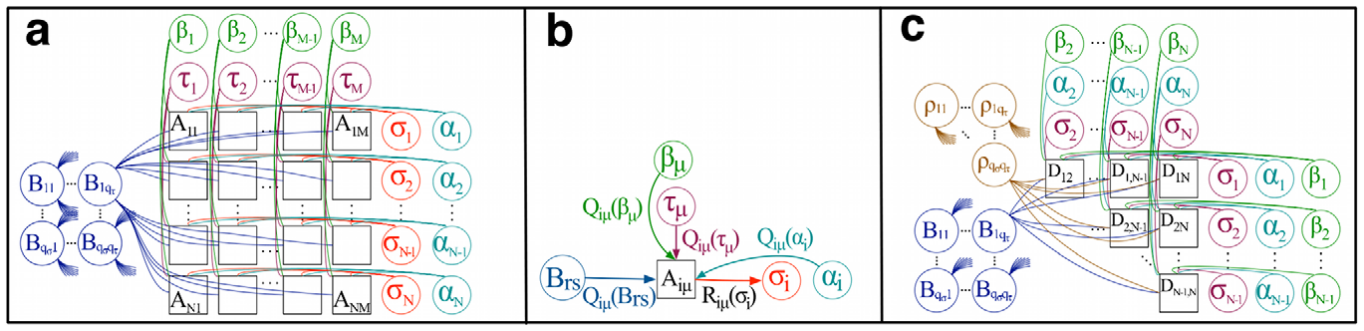
Factor graphs and an example of an elementary message passing update. Factors of the likelihood function are represented as squares, variables of the generative model as circles. Connections indicate which variables enter the calculation of which factor. **a**) For a bipartite actor-event networks represented by an 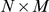 adjacency matrix 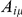, class label 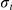 and activity 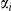 of actor 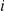 enter in the calculation of all factors in row 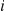. Equivalently, class label 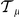 and popularity 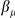 of event 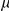 enter in the calculation of all factors in column 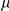. The variables 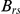 denoting preference of actors in class 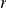 for events in class 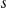 enter in every factor. Note that while each factor depends on only 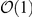 variables, the 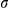 and 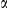 variables enter in the calculation of 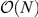, the 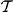 and 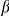 variables in 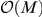 and the 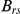 variables in 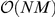 factors. **b**) Pictorial representation of the messages involved in calculating 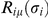 sent from factor 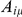 to variable 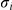 according to equation (9). **c**) For directed networks represented by non-symmetric 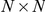 adjacency matrices, the factors correspond to dyads 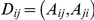. Additional to the interclass preference matrix, a symmetric matrix of reciprocities 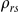 is included in the model. Every node 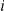 carries a single class label 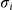, activity 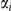 and attractiveness parameter 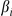. The variables associated with node 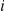 enter in the calculation of factors in both row 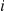 and column 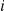.

A *message passing* or belief propagation algorithm provides a principled way to calculate approximate posterior marginal distributions [41], [42]. The starting point for this algorithm is a so-called *factor-* or *dependency-graph*, a graphical representation of the probabilistic dependencies between the variables (model parameters) we wish to infer from the data, and the individual factors that constitute the likelihood (3). Figure 5A shows this for the case of a bi-partite network, likelihood (3) and model (1).

The algorithm proceeds by exchanging messages, conditional probabilities, between factors and variables connected in the dependency graph until convergence. Using the definitions:
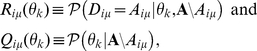
(4)


one can interpret 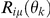 (*R-Message*) as the likelihood of a single observed matrix entry 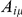 given only the parameter 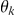 and all the data matrix except for entry 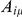. Equally, 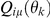 (*Q-Message*) is interpreted as the posterior probability distribution of parameter 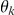 given the entire data matrix except for entry 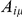. For the sake of notational economy, we have adopted to identify functions by their argument. It is to be understood that 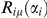 is a different function than 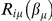 and *not* the same function 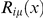 evaluated at the points 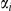 and 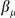 as should be clear from the definitions (4).

Formally, we obtain the R-Message from 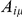 to 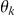, by integrating out all parameters except 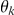 from a likelihood function
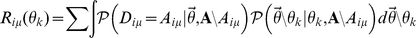
(5)


Using the independence of given data entries 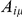 we can readily identify 
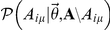
 with the 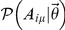 of (1). Assuming the joint distribution 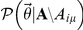 factorizes with respect to every single 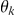, one obtains the following closed set of equations:
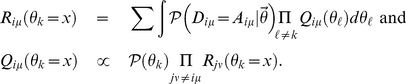
(6)


Although the factorization assumption may seem strong, it merely means that the Q-Messages 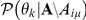 for any two variables 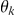 and 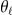 with 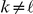 are assumed independent. Given that these distributions are conditioned on the *entire* data matrix except for one entry, the error we make using this assumption is considered negligible for large systems. The form of calculating 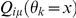 in (6) follows directly from Bayes' theorem and 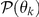 is the distribution we use to include prior information. These equations can be iterated until convergence after which we finally obtain the desired approximate marginal posterior distribution, for every single parameter, as:
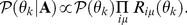
(7)


To illustrate these ideas, explicit update equations for the inference of the hidden class index 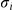 of node 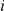 appear below. Expressions for other parameters are reported in Material S1. With
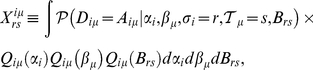
(8)


we can write for the R- and Q-Messages between 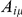 and 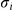:
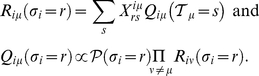
(9)


The dependency graph greatly facilitates setting-up these update equations. Following the rules that R-Messages are always sent from factors to variables and Q-Messages from variables to factors; and that in R-Messages, we sum or integrate over the incoming Q-messages, while Q-Messages are proportional to the product of incoming R-Messages, we can write the equations based on the dependency graph. Figure 5B shows a detail of 5A focussing on factor 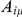 to illustrate the messages involved in the calculation of 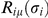 sent to variable 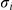 as in (9). Figure 5C illustrate the update equations in the case of directed uni-partite networks (cf. Material S1).

## Supporting Information

Material S1 **The complete update equations for learning model (1) for bi-partite networks, undirected uni-partite networks and directed uni-partite networks.** Further, it shows an example application of our method to an undirected uni-partite network, paralleling our Southern Women example in figure 2, plots of the adjacency matrix of the neural network of *c. elegans* and the model parameters estimated and used to generate the ensemble of random null models necessary for the motif analysis shown in figure 3; a description of the Newman-Leicht method [28] used in our OMIM example and matrix plots of the diseasosome network with parameter estimates as used for the generation of figure 4b.
(PDF)

Dataset S1 **The parameters estimated and the latent class assignments for the nodes of the chemical synapse network of** ***c. elegans*** **as used to generate** **figure 3** **.**
(TXT)

Dataset S2 **The gene disease associations from the OMIM database as of Dec. 2005.**
(TXT)

Dataset S3 **The gene disease associations added to the OMIM database after Dec. 2005.**
(TXT)

Dataset S4 **An example of parameter estimates and the assignments into 16 latent classes using model (1) of diseases from the OMIM database as used in** **figure 4b** **.**
(TXT)
