# Supplementary material for: The Interplay between Microscopic and Mesoscopic Structures in Complex Networks
Source: PLoS One. 2011 Aug 1;6(8):e21282. doi: 10.1371/journal.pone.0021282 (PMC3148213; doi:10.1371/journal.pone.0021282)
Supplement: Material S1 — The complete update equations for learning model (1) for bi-partite networks, undirected uni-partite networks and directed uni-partite networks. Further, it shows an example application of our method to an undirected uni-partite network, paralleling our Southern Women example in figure 2, plots of the adjacency matrix of the neural network of c. elegans and the model parameters estimated and used to generate the ensemble of random null models necessary for the motif analysis shown in figure 3; a description of the Newman-Leicht method [28] used in our OMIM example and matrix plots of the diseasosome network with parameter estimates as used for the generation of figure 4b. (PDF) [file pone.0021282.s001.pdf]

Supporting material for "The interplay between microscopic and mesoscopic structures in complex networks"

1 Update Equations

1.1 Statistical physics-based interpretation of the odds ratio

Before discussing the update equations for the specific models, we give short physical interpretation of the generative model chosen to make its exponential form obvious and show the connection of the inference problem in networks to classical disordered spin systems.

The odds ratio between the elements of the matrix $\mathbf{D}$ presented in the main text, which we reproduce here for clarity, is

$$\frac{\mathcal{P}\big(D_{i\mu}=1|\vec{\theta}\big)}{\mathcal{P}\big(D_{i\mu}=0|\vec{\theta}\big)}=\frac{\alpha_i}{(1-\alpha_i)}\frac{\beta_\mu}{(1-\beta_\mu)}\frac{B_{\sigma_i\tau_\mu}}{(1-B_{\sigma_i\tau_\mu})}. \tag{1}$$

It is possible to rewrite the above expression to obtain the probability distribution of the elements of the matrix $\mathbf{D}$ as

$$\mathcal{P}\big(D_{i\mu}|\vec{\theta}\big)\propto\exp\Big[\frac{2D_{i\mu}-1}{2}\big(a_i+b_\mu+C_{\sigma_i\tau_\mu}\big)\Big], \tag{2}$$

with

$$a_i=\ln\frac{\alpha_i}{(1-\alpha_i)}, \tag{3}$$

$$b_\mu=\ln\frac{\beta_\mu}{(1-\beta_\mu)}, \tag{4}$$

$$C_{\sigma_i\tau_\mu}=\ln\frac{C_{\sigma_i\tau_\mu}}{(1-C_{\sigma_i\tau_\mu})}. \tag{5}$$

In physics terms, as the binary matrix $\mathbf{D}$ can be considered as a spin system defined by the entries $D_{i\mu}$ [1]. The above probability distribution can then be interpreted as the Gibbs equilibrium distribution of a non-interacting system of spins with the local field $h_{i\mu}=a_i+b_\mu+C_{\sigma_i\tau_\mu}$ acting on each spin.

Obviously, a completely generic local field $h_{i\mu}$ would be equivalent to having the same number of degrees of freedom as entries in the matrix. This case would be non-informative as any data could be adjusted by defining appropriately tailored values of the local fields. From a mathematical point of view, the structure embedded in the local fields decreases the number of degrees of freedom. Physically, it can be justified by separating the influence of the fields to the different effects which depend on the row (a_i), column (b_μ) and the corresponding groups with which each of the specific elements $D_{i\mu}$ is associated ($C_{\sigma_i\tau_\mu}$).

1.2 Learning model (1) for a bi-partite network

The probabilities defined via odds ratios in (1) can be written explicitly as

$$\mathcal{P}(D_{i\mu}=x|\alpha_i,\beta_\mu,\sigma_i,\tau_\mu,\mathbf{B})=\frac{x\alpha_i\beta_\mu B_{\sigma_i\tau_\mu}+(1-x)(1-\alpha_i)(1-\beta_\mu)(1-B_{\sigma_i\tau_\mu})}{\alpha_i\beta_\mu B_{\sigma_i\tau_\mu}+(1-\alpha_i)(1-\beta_\mu)(1-B_{\sigma_i\tau_\mu})}, \tag{6}$$

where $x\in\{0,1\}$. The belief propagation algorithm operates by passing messages between factors and variables in an iterative way until convergence. For our model on a bi-partite network, the messages from factors to

variables will be given by

$$
\begin{aligned}
R_{i\mu}(\sigma_i = r) &\equiv \mathcal{P}(D_{i\mu} = A_{i\mu}|\sigma_i = r) = \sum_s X^{i\mu}_{rs} Q_{i\mu}(\tau_\mu = s), \\
R_{i\mu}(\tau_\mu = s) &\equiv \mathcal{P}(D_{i\mu} = A_{i\mu}|\tau_\mu = s) = \sum_r Q_{i\mu}(\sigma_i = r) X^{i\mu}_{rs}, \\
R_{i\mu}(\alpha_i = x) &\equiv \mathcal{P}(D_{i\mu} = A_{i\mu}|\alpha_i = x) = \sum_{rs} Q_{i\mu}(\sigma_i = r) Y^{i\mu}_{rs}(\alpha_i = x) Q_{i\mu}(\tau_\mu = s), \\
R_{i\mu}(\beta_\mu = x) &\equiv \mathcal{P}(D_{i\mu} = A_{i\mu}|\alpha_i = x) = \sum_{rs} Q_{i\mu}(\sigma_i = r) Y^{i\mu}_{rs}(\beta_\mu = x) Q_{i\mu}(\tau_\mu = s), \\
R_{i\mu}(B_{rs} = x) &\equiv \mathcal{P}(D_{i\mu} = A_{i\mu}|B_{ab} = x) \\
&= \sum_{a,b\neq r,s} Q_{i\mu}(\sigma_i = a) X^{i\mu}_{ab} Q_{i\mu}(\tau_\mu = b) + Q_{i\mu}(\sigma_i = r) Z^{i\mu}_{rs}(B_{rs} = x) Q_{i\mu}(\tau_\mu = s), \qquad (7)
\end{aligned}
$$

and messages from variables to factors by

$$
\begin{aligned}
Q_{i\mu}(\sigma_i = r) &\equiv \mathcal{P}(\sigma_i = r|\mathbf{A}\backslash A_{i\mu}) \propto \mathcal{P}(\sigma_i = r) \prod_{\nu\neq\mu} R_{i\nu}(\sigma_i = r) \\
Q_{i\mu}(\tau_\mu = s) &\equiv \mathcal{P}(\tau_\mu = s|\mathbf{A}\backslash A_{i\mu}) \propto \mathcal{P}(\tau_\mu = s) \prod_{j\neq i} R_{j\mu}(\tau_\mu = s) \\
Q_{i\mu}(\alpha_i = x) &\equiv \mathcal{P}(\alpha_i = x|\mathbf{A}\backslash A_{i\mu}) \propto \mathcal{P}(\alpha_i = x) \prod_{\nu\neq\mu} R_{i\nu}(\alpha_i = x) \\
Q_{i\mu}(\beta_\mu = x) &\equiv \mathcal{P}(\beta_\mu = x|\mathbf{A}\backslash A_{i\mu}) \propto \mathcal{P}(\beta_\mu = x) \prod_{j\neq i} R_{j\mu}(\beta_\mu = x) \\
Q_{i\mu}(B_{rs} = x) &\equiv \mathcal{P}(B_{rs} = x|\mathbf{A}\backslash A_{i\mu}) \propto \mathcal{P}(B_{rs} = x) \prod_{j\nu\neq i\mu} R_{j\nu}(B_{rs} = x), \qquad (8)
\end{aligned}
$$

where

$$
\begin{aligned}
X^{i\mu}_{rs} &\equiv \int d\alpha_i d\beta_\mu dB_{rs} \mathcal{P}(D_{i\mu} = A_{i\mu}|\alpha_i, \beta_\mu, \sigma_i = r, \tau_\mu = s, B_{rs}) \times \\
&\quad Q_{i\mu}(\alpha_i) Q_{i\mu}(\beta_\mu) Q_{i\mu}(B_{rs}), \\
Y^{i\mu}_{rs}(\alpha_i = x) &\equiv \int d\beta_\mu dB_{rs} \mathcal{P}(D_{i\mu} = A_{i\mu}|\alpha_i = x, \beta_\mu, \sigma_i = r, \tau_\mu = s, B_{rs}) \times \\
&\quad Q_{i\mu}(\beta_\mu) Q_{i\mu}(B_{rs}), \\
Y^{i\mu}_{rs}(\beta_\mu = x) &\equiv \int d\alpha_i dB_{rs} \mathcal{P}(D_{i\mu} = A_{i\mu}|\alpha_i, \beta_\mu = x, \sigma_i = r, \tau_\mu = s, B_{rs}) \times \\
&\quad Q_{i\mu}(\alpha_i) Q_{i\mu}(B_{rs}), \\
Z^{i\mu}_{rs}(B_{rs} = x) &\equiv \int d\alpha_i d\beta_\mu \mathcal{P}(D_{i\mu} = A_{i\mu}|\alpha_i, \beta_\mu, \sigma_i = r, \tau_\mu = s, B_{rs} = x) \times \\
&\quad Q_{i\mu}(\alpha_i) Q_{i\mu}(\beta_\mu). \qquad (9)
\end{aligned}
$$

These integrations can be done numerically, keeping in mind that the range of all variables α_i, β_μ and B_{rs} is only $(0,1)$. However, since $\mathcal{P}\big(D_{i\mu} = A_{i\mu}|\vec{\theta}\big)$ is generally weakly dependent on any individual parameter, if the distributions $Q_{i\mu}(\theta_k)$ have small to moderate variance, an expansion around the expectation values of θ_k under $Q_{i\mu}(\theta_k)$ to zeroth order gives an excellent approximation. With the mean values calculated as

$$
\begin{aligned}
\alpha^{i\mu} &\equiv \int \alpha_i Q_{i\mu}(\alpha_i) d\alpha_i, \\
\beta^{i\mu} &\equiv \int \beta_\mu Q_{i\mu}(\beta_\mu) d\beta_\mu, \\
B^{i\mu}_{rs} &\equiv \int B_{rs} Q_{i\mu}(B_{rs}) dB_{rs}, \qquad (10)
\end{aligned}
$$

we can simply insert these values into (1) to obtain

$$
\begin{aligned}
X_{rs}^{i\mu} &\approx \mathcal{P}\big(D_{i\mu} = A_{i\mu}|\alpha^{i\mu}, \beta^{i\mu}, \sigma_i = r, \tau_\mu = s, B_{rs}^{i\mu}\big),\\
Y_{rs}^{i\mu}(\alpha_i = x) &\approx \mathcal{P}\big(D_{i\mu} = A_{i\mu}|\alpha_i = x, \beta^{i\mu}, \sigma_i = r, \tau_\mu = s, B_{rs}^{i\mu}\big),\\
Y_{rs}^{i\mu}(\beta_\mu = x) &\approx \mathcal{P}\big(D_{i\mu} = A_{i\mu}|\alpha^{i\mu}, \beta_\mu = x, \sigma_i = r, \tau_\mu = s, B_{rs}^{i\mu}\big),\\
Z_{rs}^{i\mu}(B_{rs} = x) &\approx \mathcal{P}\big(D_{i\mu} = A_{i\mu}|\alpha^{i\mu}, \beta^{i\mu}, \sigma_i = r, \tau_\mu = s, B_{rs} = x\big).
\end{aligned}
\tag{11}
$$

After convergence, the posterior marginals can be calculated from the resulting values of the messages as

$$
\begin{aligned}
\mathcal{P}(\sigma_i = r|\mathbf{A}) &\propto \mathcal{P}(\sigma_i = r)\prod_\mu R_{i\mu}(\sigma_i = r),\\
\mathcal{P}(\tau_\mu = s|\mathbf{A}) &\propto \mathcal{P}(\tau_\mu = s)\prod_i R_{i\mu}(\tau_\mu = s),\\
\mathcal{P}(\alpha_i = x|\mathbf{A}) &\propto \mathcal{P}(\alpha_i = x)\prod_\mu R_{i\mu}(\alpha_i = x),\\
\mathcal{P}(\beta_\mu = x|\mathbf{A}) &\propto \mathcal{P}(\beta_\mu = x)\prod_i R_{i\mu}(\beta_\mu = x),\\
\mathcal{P}(B_{rs} = x|\mathbf{A}) &\propto \mathcal{P}(B_{rs} = x)\prod_{i\mu} R_{i\mu}(B_{rs} = x).
\end{aligned}
\tag{12}
$$

We see that at any point in time during the update sequence of the messages, the Q-Messages differ from the current estimate of the posterior marginals only by a single R-Message. Since each variable receives a very large number of these messages, we can conclude that the Q-messages depend only weakly on which R-Message is left out. We may safely assume that the means of the variables in (10) will depend very weakly on μ for $\alpha^{i\mu}$, on i for $\beta^{i\mu}$ and on i, μ for $B_{rs}^{i\mu}$ such that each can be approximated by a single value. This reduces the computational effort considerably. The same reasoning applies in the cases of uni-partite networks.

The computational complexity for an update of a single variable σ_i is $\mathcal{O}(Mq_\sigma q_\tau)$ where q_σ and q_τ are the maximum number of classes allowed for σ_i and τ_μ, respectively, *i. e.* the size of the matrix $\mathbf{B}$. Since there are N variables σ_i to update, the total computational complexity scales as $\mathcal{O}(NMq_\sigma q_\tau)$, *i. e.* linear in the size of the adjacency matrix and linear in the size of the model. The same complexity is achieved for the updates of the α_i, τ_μ, β_μ and B_{rs} variables. Convergence is generally reached in less than 100 update sweeps over all variables.

1.3 Update equations when learning model (1) for an undirected, uni-partite network

In the case of undirected networks, we cannot differentiate between activity and popularity and hence we drop the set of parameters β_μ. The model probability of two nodes being connected or not then reads as:

$$\mathcal{P}(D_{ij} = x|\alpha_i, \alpha_j, \sigma_i, \sigma_j, \mathbf{B}) = \frac{x\alpha_i\alpha_j B_{\sigma_i\sigma_j} + (1-x)(1-\alpha_i)(1-\alpha_j)(1-B_{\sigma_i\sigma_j})}{\alpha_i\alpha_j B_{\sigma_i\sigma_j} + (1-\alpha_i)(1-\alpha_j)(1-B_{\sigma_i\sigma_j})}, \tag{13}$$

where again $x \in \{0,1\}$. Using the same arguments as before, we can write the following definitions analogous to (11)

$$\begin{aligned} X_{rs}^{ij} &\approx \mathcal{P}\big(D_{ij} = A_{ij}|\alpha^{ij}, \alpha^{ji}, \sigma_i = r, \sigma_j = s, B_{rs}^{i\mu}\big), \\ Y_{rs}^{ij}(\alpha_i = x) &\approx \mathcal{P}\big(D_{ij} = A_{ij}|\alpha_i = x, \alpha^{ji}, \sigma_i = r, \sigma_j = s, B_{rs}^{ij}\big), \\ Z_{rs}^{ij}(B_{rs} = x) &\approx \mathcal{P}\big(D_{ij} = A_{ij}|\alpha^{ij}, \alpha^{ji}, \sigma_i = r, \sigma_j = s, B_{rs} = x\big), \end{aligned} \tag{14}$$

which lead to update equations of the form

$$\begin{aligned} R_{ij}(\sigma_i = r) &\equiv \mathcal{P}(D_{ij} = A_{ij}|\sigma_i = r) = \sum_s X_{rs}^{ij} Q_{ij}(\sigma_j = s), \\ R_{ij}(\alpha_i = x) &\equiv \mathcal{P}(D_{ij} = A_{ij}|\alpha_i = x) = \sum_{rs} Q_{ij}(\sigma_i = r) Y_{rs}^{ij}(\alpha_i = x) Q_{ij}(\sigma_j = r) \\ R_{ij}(B_{rs} = x) &\equiv \mathcal{P}(D_{ij} = A_{ij}|B_{ab} = x), \\ &= \sum_{a,b \neq r,s} Q_{ij}(\sigma_i = a) X_{ab}^{ij} Q_{ij}(\sigma_j = b) + Q_{ij}(\sigma_i = r) Z_{rs}^{ij}(B_{rs} = x) Q_{ij}(\sigma_j = s), \end{aligned} \tag{15}$$

$$\begin{aligned} Q_{ij}(\sigma_i = r) &\equiv \mathcal{P}(\sigma_i = r|\mathbf{A}\backslash A_{ij}) \propto \mathcal{P}(\sigma_i = r) \prod_{k \neq j} R_{ik}(\sigma_i = r), \\ Q_{ij}(\alpha_i = x) &\equiv \mathcal{P}(\alpha_i = x|\mathbf{A}\backslash A_{ij}) \propto \mathcal{P}(\alpha_i = x) \prod_{k \neq j} R_{ik}(\alpha_i = x), \\ Q_{ij}(B_{rs} = x) &\equiv \mathcal{P}(B_{rs} = x|\mathbf{A}\backslash A_{ij}) \propto \mathcal{P}(B_{rs} = x) \prod_{kl \neq ij} R_{kl}(B_{rs} = x). \end{aligned} \tag{16}$$

1.4 Update equations when learning a model from a directed, uni-partite network

When studying directed networks, we have to take into account that a link from node i to node j is generally not statistically independent from the link from node j to node i. The tendency for reciprocation can can be modeled explicitly by introducing an additional parameter ρ, the value of which can be inferred from the data alone.

In contrast to the models discussed so far, for directed networks the likelihood function does not factor into terms corresponding to single links, but into terms corresponding to pairs of nodes:

$$\mathcal{L}(\vec{\theta}) \equiv \mathcal{P}\big(\mathbf{A}|\vec{\theta}\big) = \prod_{i<j} \mathcal{P}\big(D_{ij} = (A_{ij}, A_{ji})|\vec{\theta}\big). \tag{17}$$

The individual dyad D_{ij} can assume four different values: $D_{ij} = (1,1)$, corresponding to a reciprocated link between nodes i and j, $D_{ij} = (1,0)$, corresponding to a non-reciprocated link between nodes i and j, $D_{ij} = (0,1)$, corresponding to a non-reciprocated link between nodes j and i, and $D_{ij} = (0,0)$, corresponding an unconnected pair of nodes i and j.

The explicit expression for the probability of a particular dyad configuration can be written using the abbreviation

$$\mathcal{C}_{ij}(x) = (\alpha_i \beta_j B_{\sigma_i\sigma_j})^x \left((1-\alpha_i)(1-\beta_j)(1-B_{\sigma_i\sigma_j})\right)^{(1-x)}, \tag{18}$$

as

$$\mathcal{P}(D_{ij} = (x,y)|\alpha_i, \beta_i, \alpha_j, \beta_j, \sigma_i, \sigma_j, \rho, \mathbf{B}) = \frac{\rho_{\sigma_i\sigma_j}^{xy}(1-\rho_{\sigma_i\sigma_j})^{(1-xy)}\mathcal{C}_{ij}(x)\,\mathcal{C}_{ji}(y)}{\sum_{a,b=0}^{1}\rho_{\sigma_i\sigma_j}^{ab}(1-\rho_{\sigma_i\sigma_j})^{(1-ab)}\mathcal{C}_{ij}(x)\,\mathcal{C}_{ji}(y)}, \tag{19}$$

where $x, y \in \{0,1\}$ and we restrict ourselves to networks without selfloops, *i. e.* we consider only the the index pairs (i,j) with $i < j$. Further, we have allowed the reciprocity parameter ρ_{rs} to depend explicitly on the latent classes of nodes we infer. Hence, we allow reciprocity to vary between different latent classes of nodes. We believe this to be a natural assumption.

We will make the same simplifying assumptions as in model (1) with respect to the simple calculation of the terms X, Y and Z. In addition to the means already defined in (10), we introduce

$$\rho_{rs}^{ij} \equiv \int \rho_{rs} Q_{ij}(\rho_{rs}) d\rho_{rs}, \tag{20}$$

as the mean value of the reciprocity in block r, s. Analogous to (11), we then write

$$\begin{aligned}
X_{rs}^{ij} &\approx \mathcal{P}\big(D_{ij} = (A_{ij}, A_{ji}) | \alpha^{ij}, \beta^{ij}, \alpha^{ij}, \beta^{ji}, \sigma_i = r, \sigma_j = s, B_{rs}^{ij}, \rho_{rs}^{ij}\big), \\
Y_{rs}^{ij}(\alpha_i = x) &\approx \mathcal{P}\big(D_{ij} = (A_{ij}, A_{ji}) | \alpha_i = x, \beta^{ij}, \alpha^{ji}, \beta^{ji}, \sigma_i = r, \sigma_j = s, B_{rs}^{ij}, \rho_{rs}^{ij}\big) \\
Y_{rs}^{ij}(\beta_i = x) &\approx \mathcal{P}\big(D_{ij} = (A_{ij}, A_{ji}) | \alpha^{ij}, \beta_i = x, \alpha^{ji}, \beta^{ji}, \sigma_i = r, \sigma_j = s, B_{rs}^{ij}, \rho_{rs}^{ij}\big), \\
Y_{rs}^{ij}(\alpha_j = x) &\approx \mathcal{P}\big(D_{ij} = (A_{ij}, A_{ji}) | \alpha_{ij}, \beta^{ij}, \alpha_j = x, \beta^{ji}, \sigma_i = r, \sigma_j = s, B_{rs}^{ij}, \rho_{rs}^{ij}\big) \\
Y_{rs}^{ij}(\beta_j = x) &\approx \mathcal{P}\big(D_{ij} = (A_{ij}, A_{ji}) | \alpha^{ij}, \beta_{ij}, \alpha^{ji}, \beta_j = x, \sigma_i = r, \sigma_j = s, B_{rs}^{ij}, \rho_{rs}^{ij}\big), \\
Z_{rs}^{ij}(\rho_{rs} = x) &\approx \mathcal{P}\big(D_{ij} = (A_{ij}, A_{ji}) | \alpha^{ij}, \beta^{ij}, \alpha^{ji}, \beta^{ji}, \sigma_i = r, \sigma_j = s, B_{rs}^{ij}, \rho_{rs} = x\big) \\
Z_{rs}^{ij}(B_{rs} = x) &\approx \mathcal{P}\big(D_{ij} = (A_{ij}, A_{ji}) | \alpha^{ij}, \beta^{ij}, \alpha^{ji}, \beta^{ji}, \sigma_i = r, \sigma_j = s, B_{rs} = x, \rho_{rs}^{ij}\big).
\end{aligned} \tag{21}$$

With these values calculated, the complete set of update equations becomes:

$$\begin{aligned}
R_{ij}(\sigma_i = r) &\equiv \mathcal{P}(D_{ij} = (A_{ij}, A_{ji}) | \sigma_i = r) = \sum_s X_{rs}^{ij} Q_{ij}(\sigma_j = s), \\
R_{ij}(\alpha_i = x) &\equiv \mathcal{P}(D_{ij} = (A_{ij}, A_{ji}) | \alpha_i = x) = \sum_{rs} Q_{ij}(\sigma_i = r) Y_{rs}^{ij}(\alpha_i = x) Q_{ij}(\sigma_j = s), \\
R_{ij}(\beta_i = x) &\equiv \mathcal{P}(D_{ij} = (A_{ij}, A_{ji}) | \beta_i = x) = \sum_{rs} Q_{ij}(\sigma_i = r) Y_{rs}^{ij}(\beta_i = x) Q_{ij}(\sigma_j = s), \\
R_{ij}(\alpha_j = x) &\equiv \mathcal{P}(D_{ij} = (A_{ij}, A_{ji}) | \alpha_j = x) = \sum_{rs} Q_{ij}(\sigma_i = r) Y_{rs}^{ij}(\alpha_j = x) Q_{ij}(\sigma_j = s), \\
R_{ij}(\beta_j = x) &\equiv \mathcal{P}(D_{ij} = (A_{ij}, A_{ji}) | \beta_j = x) = \sum_{rs} Q_{ij}(\sigma_i = r) Y_{rs}^{ij}(\beta_j = x) Q_{ij}(\sigma_j = s), \\
R_{ij}(\rho_{rs} = x) &\equiv \mathcal{P}(D_{ij} = (A_{ij}, A_{ji}) | B_{ab} = x) \\
&= \sum_{a,b \neq r,s} Q_{ij}(\sigma_i = a) X_{ab}^{ij} Q_{ij}(\sigma_j = b) + Q_{ij}(\sigma_i = r) Z_{rs}^{ij}(\rho_{rs} = x) Q_{ij}(\sigma_j = s), \\
R_{ij}(B_{rs} = x) &\equiv \mathcal{P}(D_{ij} = (A_{ij}, A_{ji}) | B_{ab} = x) \\
&= \sum_{a,b \neq r,s} Q_{ij}(\sigma_i = a) X_{ab}^{ij} Q_{ij}(\sigma_j = b) + Q_{ij}(\sigma_i = r) Z_{rs}^{ij}(B_{rs} = x) Q_{ij}(\sigma_j = s),
\end{aligned} \tag{22}$$

$$\begin{aligned}
Q_{ij}(\sigma_i = r) &\equiv \mathcal{P}(\sigma_i = r | \mathbf{A} \backslash A_{ij}) \propto \mathcal{P}(\sigma_i = r) \prod_{i<k} R_{ik}(\sigma_i = r) \prod_{l<i} R_{li}(\sigma_i = r), \\
Q_{ij}(\alpha_i = x) &\equiv \mathcal{P}(\alpha_i = x | \mathbf{A} \backslash A_{ij}) \propto \mathcal{P}(\alpha_i = x) \prod_{i<k} R_{ik}(\alpha_i = x) \prod_{l<i} R_{li}(\alpha_i = x), \\
Q_{ij}(\beta_i = x) &\equiv \mathcal{P}(\beta_i = x | \mathbf{A} \backslash A_{ij}) \propto \mathcal{P}(\beta_i = x) \prod_{i<k} R_{ik}(\beta_i = x) \prod_{l<i} R_{li}(\beta_i = x), \\
Q_{ij}(\rho_{rs} = x) &\equiv \mathcal{P}(\rho_{rs} = x | \mathbf{A} \backslash A_{ij}) \propto \mathcal{P}(\rho_{rs} = x) \prod_{k<l, kl \neq ij} R_{kl}(\rho_{rs} = x), \\
Q_{ij}(B_{rs} = x) &\equiv \mathcal{P}(B_{rs} = x | \mathbf{A} \backslash A_{ij}) \propto \mathcal{P}(B_{rs} = x) \prod_{k<l, kl \neq ij} R_{kl}(B_{rs} = x),
\end{aligned} \tag{23}$$

2 Structural analysis of undirected, uni-partite networks

As an example of inferring class structure in an undirected, unipartite network, we study another well known social network: the Zachary Karate Club [2]. In the 1970's, American sociologist investigated the social interactions among the 34 members of a karate club at an American university. The social network recorded is shown in figure 1. Over the course of the study, an argument between the instructor (node 1) and the manager (node 34) resulted in the split up of the club. The members siding with the manager in the conflict are drawn as circles in figure 1, while those siding with the instructor are represented as squares. The network has become a standard test case of "community detection" algorithms over the years, in particular after the publication of [3]. However, community detection algorithms already make one strong assumption about the network's structure: they suppose from the start that the network consists of groups nodes which are densely connected internally and only sparsely connected between groups, *i. e.* they *presuppose* the existence of the very structure they search for. In the light of this, it may actually not be surprising that community detection algorithms perform very well at "inferring" the communities the club split into, since community detection only search for a good split of the network, effectively assuming a diagonal preference matrix B_{rs} from the start. The stochastic block models are much more impartial in this respect as they allow a much richer topology between classes.

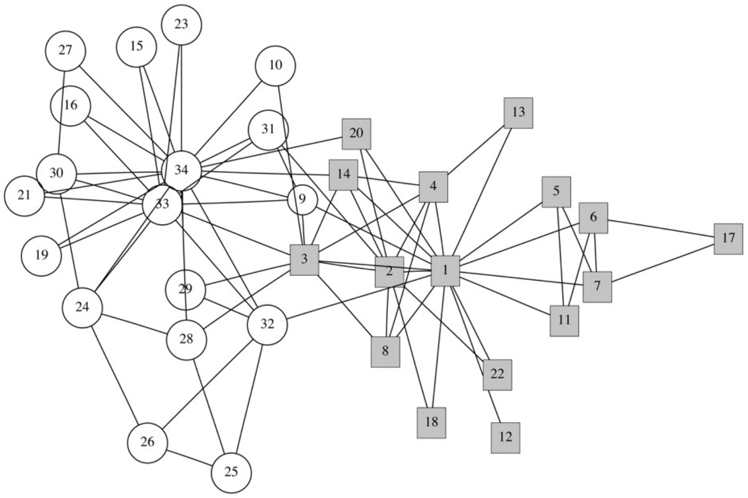

Figure 1: Network representation of Zachary's Karate Club. Figure from [3].

When now estimating latent classes using a classic stochastic block model, we find a structure that is remarkably different from the community structure so often cited for this club. Figures 2A and 2B show that analyzing the data and estimating a model that included group effects only (model (2) in the manuscript), we find a core-periphery structure, rather than a community structure! There are five core-members, including the instructor and the manager of the club, which have many interactions with the rest of the club members, while the remaining club members interact rather sparsely among each other. If we did not have the additional information about the break-up of the club, then we could be perfectly content with the interpretation that the social network of the club corresponding to its organizational structure, with manager and instructor plus a few members at the center and the rest of the members in peripheral positions.

On the other hand, when estimating model (1) from the manuscript and explicitly including parameters to model individual node activity in the class structure inference, then we can recover the observed split of the club as a community structure as shown in 2C and 2D. Note that this time, we explicitly have *not* assumed a community structure to search for. The model is still free to choose whatever preference matrix the data may

support. Again, we see that including individual node effects markedly increases the agreement between class structure inferred from network data and that observed based on domain information.

The example provides also a visual explanation for the way the message passing algorithm implements an Occam's razor. Comparing the posterior densities for the preference matrix B_{rs} in figures 2B and 2C, we observe that larger classes allow for sharper estimates of B_{rs}

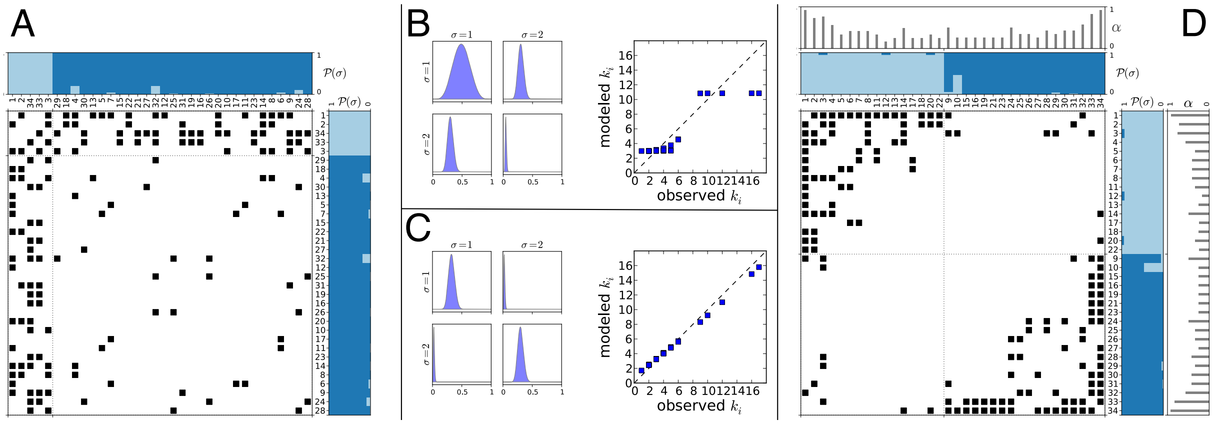

Figure 2: Comparison of structure detected in Zachary's Karate Club Network. **A** When inferring a stochastic block model with 2 classes including only group effects (as in equation (2) in the manuscript), we find a core-periphery structure, with the class assignment of nodes dominated by the different degrees. **B** Posterior densities for the entries of the preference matrix B_{rs} and expected vs. observed degrees in the model. **C** When including node specific effects in the model estimated (as in equation (1) in the manuscript), the preference matrix B_{rs} is inferred as being practically diagonal and the observed degrees distribution can be captured in the model. **D** The adjacency matrix ordered according to the classes inferred using model (1) from the manuscript. The class assignments found correspond almost perfectly to that observed in the real data, except for node 10 which cannot be assigned into one of the two classes with certainty based on the data. Comparing with figure 1, we note that the connectivity of node 10 does in fact provide little evidence for a clear class assignment based on the network alone.

3 Motif distribution in the C. elegans chemical synapse network

The box plots in figure 3c of the manuscript were obtained from motif counts in an ensemble of random networks generated using model (19) with parameters estimated from the chemical synapse network of C. elegans and allowing for 15 latent classes of nodes. The following figures 3 and 4 then show the adjacency matrix of the C. elegans chemical synapse network together with complete posterior distributions of the estimated latent classes $\mathcal{P}(\sigma_i|\mathbf{A})$, the posterior means of the estimated α_i and β_i as well as the posterior distributions for the estimated reciprocity ρ and preference matrix $\mathbf{B}$. This allows for an assessment of the model fit as well as an overview of the parameters estimated. Detailed information on the class assignment of each neuron can be found in the file `Dataset S1` included with the supporting material.

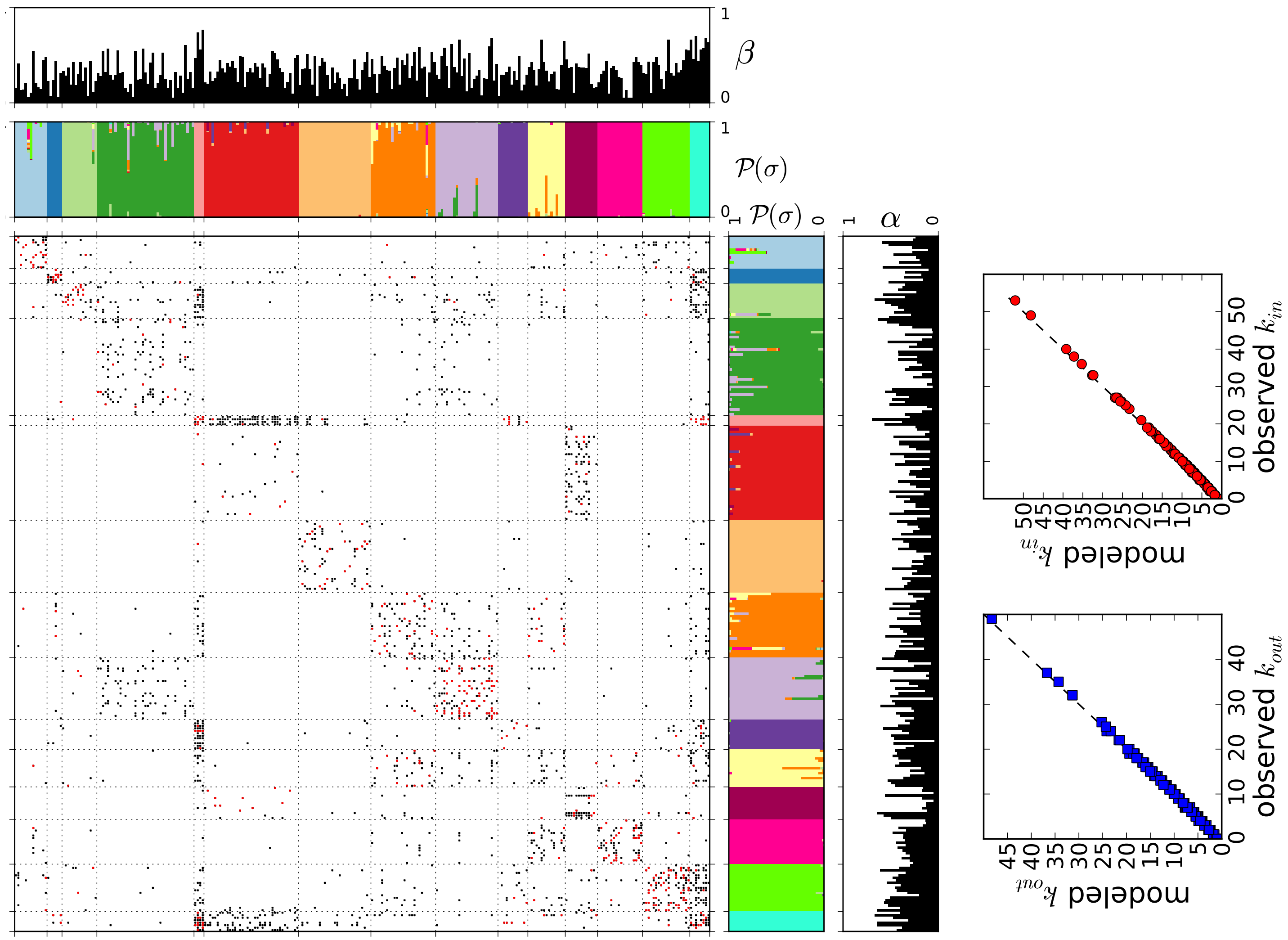

Figure 3: **Left** The *C. elegans* chemical synapse network and posterior probabilities of class assignments and mean estimates for α_i and β_i in a 15 class model according to equation (19). Rows and columns of the incidence matrix are ordered such that nodes with the same maximum component in $\mathcal{P}(\sigma_i|\mathbf{A})$ are adjacent. Reciprocated links between neurons i and j are drawn in red, non-reciprocated in black. Note the high convergence of the posterior probability of class assignments onto single classes for almost all nodes, indicating a very good model fit. **Right** Expected vs. observed in- and out-degree for this network under the same model. The expected degree sequence for both in- and out-degrees matches the observed sequence almost perfectly.

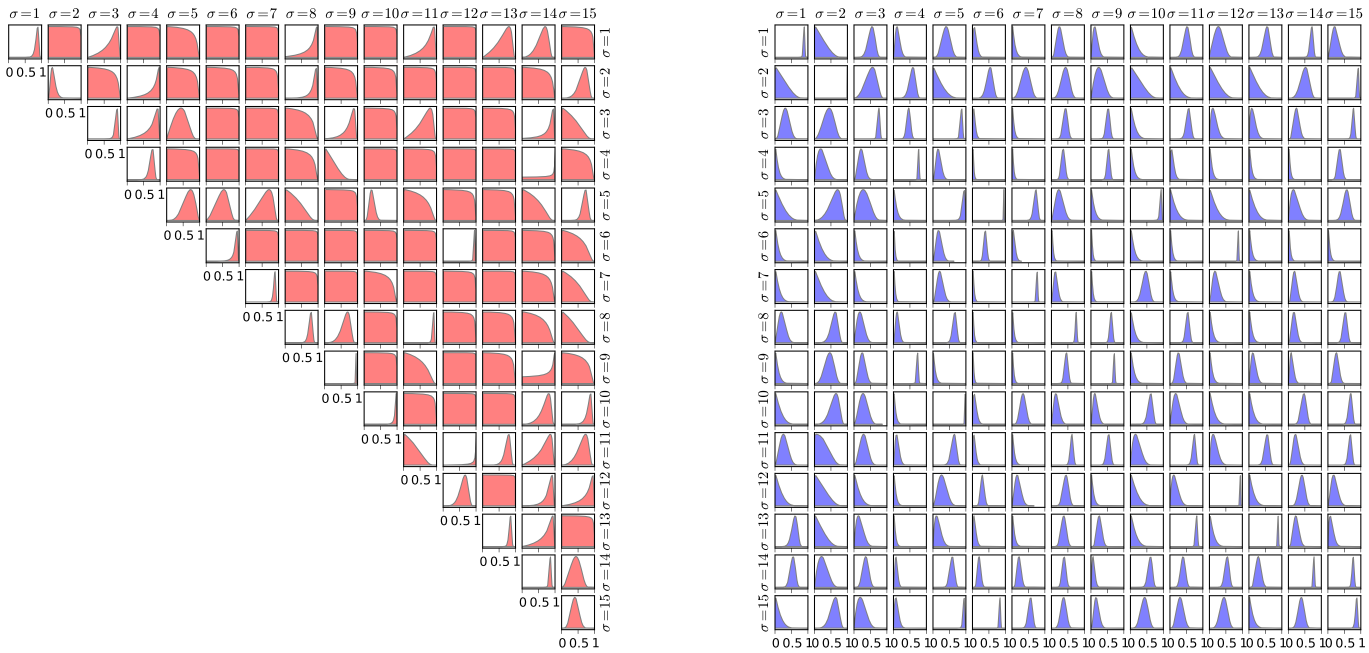

Figure 4: Posterior densities for ρ_{rs} and B_{rs} estimated from the *C. elegans* chemical synapse network using a model with 15 classes. **Left** Posterior densities for the reciprocity parameter ρ_{rs}. Note how the density is practically flat when there are no, or only very few links between nodes, *e. g.* between classes 1 and 2. Since $\rho_{rs} = \rho_{sr}$, only the upper triangle of ρ is shown. **Right** Posterior densities for the preference matrix B_{rs}. Note how the posterior is sharper when the estimate is based on connections between larger classes (compare figure 3) and the asymmetry in synaptic network is also captured in an asymmetric preference matrix.

4 Genes and diseases in the Diseasosome-Network

To obtain figure 4A, we learned both models (1), (2) and the Newman/Leicht (NL) model [4] from the diseasosome network published in [5]. The network contains the associations of genes and diseases from the Online Mendelian Inheritance in Man (OMIM) morbidmap labeled with the highest confidence tag of "3" as of December 2005. Reference [5], provided an expert classification of the diseases into 22 classes, two of which, however, were "multiple" and "undefined". Therefore, we restricted our models to contain no more than 20 classes. The NL model was originally specified for uni-partite networks. Adaptation to bipartite networks, however, is straight forward. We here give the update formulas of the expectation maximization algorithm used [4]:
E-Step:

$$q_{i\sigma} = \frac{\pi_\sigma \prod_\mu \theta_{\sigma\mu}^{A_{i\mu}}}{\sum_{\sigma'} \pi_{\sigma'} \prod_\mu \theta_{\sigma'\mu}^{A_{i\mu}}} \text{ and } q_{\mu\tau} = \frac{\pi_\tau \prod_i \theta_{\tau i}^{A_{i\mu}}}{\sum_{\tau'} \pi_{\tau'} \prod_i \theta_{\tau' i}^{A_{i\mu}}} \tag{24}$$

M-Step:

$$\begin{aligned} \pi_\sigma &= \frac{1}{N}\sum_i q_{i\sigma} \text{ and } \pi_\tau = \frac{1}{M}\sum_\mu q_{\mu\tau} \\ \theta_{\sigma\mu} &= \frac{\sum_i A_{i\mu} q_{i\sigma}}{\sum_{i'} k_i' q_{i'\sigma}} \text{ and } \theta_{\tau i} = \frac{\sum_\mu A_{i\mu} q_{\mu\tau}}{\sum_{\mu'} k_{\mu'} q_{\mu'\tau}} \end{aligned} \tag{25}$$

Corresponding to model (1), we consider $\mathbf{A}$ as an actor-event matrix. The possible latent classes for actors (rows) i are indexed with σ and for events (columns) μ with τ. Following the notation introduced in [4], π_σ then is the probability that any randomly chosen actor belongs to class σ and π_τ is the probability that any randomly chosen event belongs to class τ. Further, $q_{i\sigma}$ is the probability that a specific actor i belongs to class σ and $q_{\mu\tau}$ is the probability that a specific event μ belongs to class τ. Finally, $\theta_{\sigma\mu}$ is the probability that event μ is attended by any actor of class σ, *i. e.* the "popularity" of event μ among the members of class σ and $\theta_{\tau i}$ is the probability that actor i attends any event of class τ, *i. e.* the "activity" of actor i with respect to events in class τ. Note how these θ-parameters are class specific as compared to the global popularities $\beta\mu$ and activities α_i of model (1). Initialization and update schedule was the same as in Ref. [4]. The probability of a single matrix entry is then given up to a constant factor as $\mathcal{P}(D_{i\mu} = 1|\vec{\theta}) \propto \sum_{\sigma\tau} \pi_\sigma q_{i\sigma} \theta_{\sigma\mu} \pi_\tau q_{\mu\tau} \theta_{\tau i}$.

For figure 4A, for each number of classes in the model, we choose the best (in terms of maximum likelihood of the data under the parameters estimated) of 10 runs with different random initial conditions for models (1) and (2) and the best of 50 runs with different random initial conditions for the NL model.

For figure 4B, we used the parameters estimated from best runs with 16 classes shown in figure 4A for all three models. One candidate list ranking the possible associations was hence obtained for each model. Conclusions from figure 4B are valid for other numbers of classes as well.

The following figure 5 shows the raw data including the posterior probabilities of class assignment and maxima of posterior density of α_i and β_μ for model (1) with 16 classes. The raw data can be found in the file `Dataset S2` and additions to the database in the file `Dataset S3`. The complete assignment of diseases into the 16 classes as shown in the example can be found in the file `Dataset S4`.

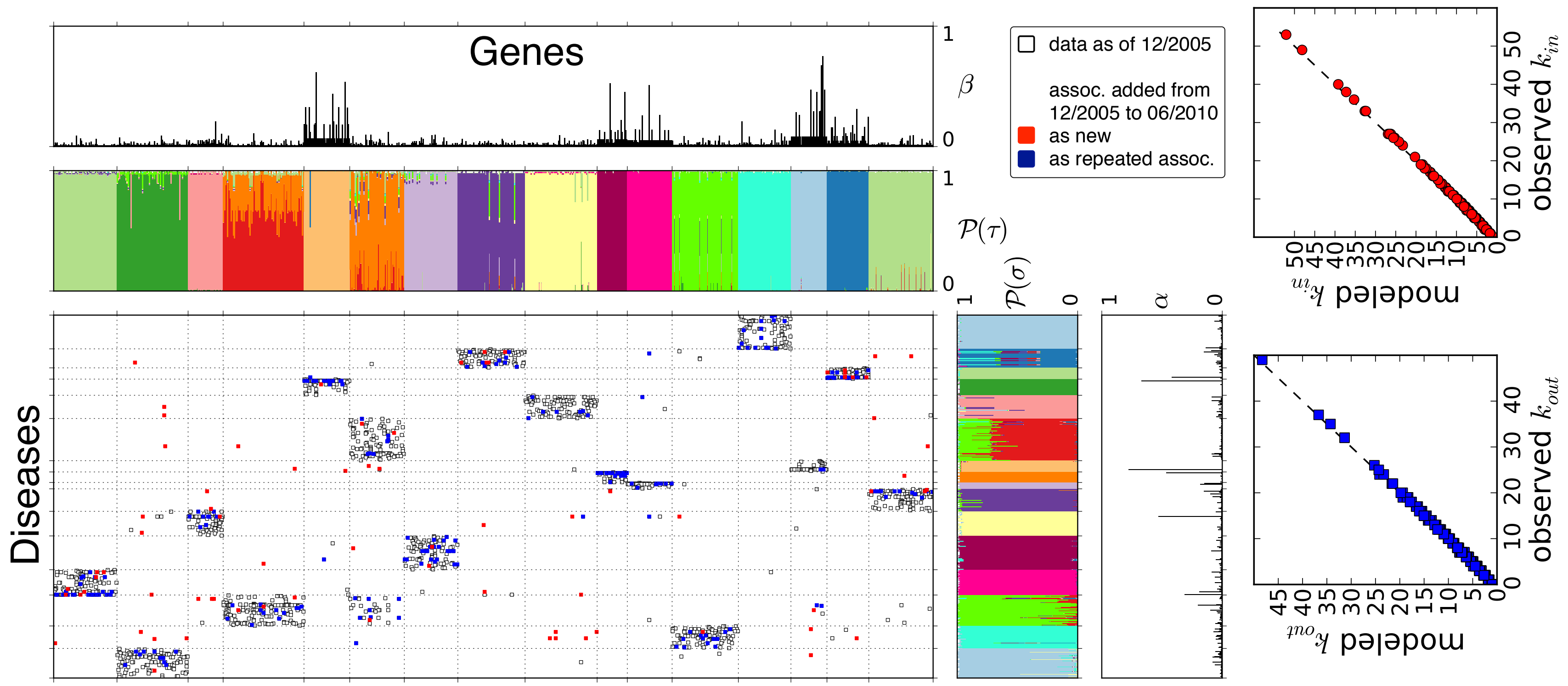

Figure 5: The gene-disease association matrix with rows and columns reordered according to classes inferred by estimating the parameters of model (1) with 16 classes from the data available by Dec. 2005. Blue and red matrix entries mark associations added to the database between Dec. 2005 and Jun. 2010, with blue indicating new variants of already entered diseases and red marking genuinely new associations.

References

[1] H. Nishimori. *Statistical Physics of Spin Glasses and Information Processing*. Oxford Science Publications, Oxford, 2001.

[2] W. Zachary. An information flow model for conflict and fission in small groups. *J. Anthr. Res.*, 33:452–473, 1977.

[3] M. E. J. Newman and M. Girvan. Finding and evaluating community structure in networks. *Phys. Rev. E*, 69:026113, 2004.

[4] M.E.J. Newman and E.A. Leicht. Mixture models and exploratory data analysis in networks. *Proc Natl Acad Sci USA*, 104(23):9564–9569, 2007.

[5] Kwang-Il Goh, Michael E. Cusick, David Valle, Barton Childs, Mark Vidal, and Albert-László Barabási. The human disease network. *Proc Natl Acad Sci USA*, 104(21):8685–8690, 2007.
